# Clinical predictive factors of the efficacy of immune checkpoint inhibitors and kinase inhibitors in advanced hepatocellular cancer

**DOI:** 10.1007/s12094-024-03644-9

**Published:** 2024-08-19

**Authors:** Yunyun Lu, Yi Lu

**Affiliations:** Department of Radiation Oncology, Ningbo Medical Center Lihuili Hospital, Ningbo, 315048 Zhejiang China

**Keywords:** Hepatocellular carcinoma, Immune checkpoint inhibitor, Kinase inhibitor, Platelet-to-lymphocyte ratio, Total cholesterol

## Abstract

**Background:**

Hepatocellular carcinoma (HCC) is a highly aggressive tumor associated with significant morbidity and mortality rates. Combination therapy with immune checkpoint inhibitors (ICIs) and kinase inhibitors has emerged as a promising strategy for liver cancer treatment in recent years. However, the clinical factors predicting the outcomes of combination therapy in patients with advanced liver cancer remain uncertain. Therefore, this study investigated the relationships between clinical predictors and the efficacy of ICI plus kinase inhibitor therapy to personalize treatment plans.

**Methods:**

We retrospectively enrolled 98 patients who received combination treatment with ICIs and kinase inhibitors for advanced HCC. Based on blood lipid levels and other clinical factors prior to treatment, we investigated potential biomarkers that could predict treatment responses in this patient population.

**Results:**

Mean progression-free survival (PFS) and overall survival (OS) in this cohort were 10.1 and 17.2 months, respectively. Via multivariate analysis, the absence of extrahepatic metastasis, the absence of portal vein thrombosis (PVT), neutrophil-to-lymphocyte ratio (NLR) < 3.225, platelet-to-lymphocyte ratio (PLR) < 140.75, and prognostic nutritional index (PNI) ≥ 37.25 were identified as independent predictors of improved PFS. Factors associated with better OS included PLR < 140.75 and total cholesterol (TC) < 3.46 mmol/L. Univariate analysis identified significant associations of Eastern Cooperative Oncology Group performance status (ECOG PS), hepatitis B virus (HBV) DNA levels, Child–Pugh classification, alpha-fetoprotein (AFP), TC, and the receipt of regorafenib with PFS. Additionally, ECOG PS, Child–Pugh classification, AFP, PVT, NLR, PNI, and the receipt of regorafenib were significantly associated with OS.

**Conclusions:**

PLR and TC were potential clinical predictive factors for survival outcomes in patients with advanced HCC who received ICI/kinase inhibitor combination therapy. It is important to know the clinical characteristics of patients prior to treatment initiation to optimize outcomes.

## Introduction

Liver cancer is the second leading cause of cancer death in China, and its mortality rate has significantly increased in the US [1]. Approximately 85–90% of patients with liver cancer have primary hepatocellular carcinoma (HCC). Various factors such as metabolic problems, infectious diseases including hepatitis, and lifestyle factors including alcoholism and smoking contribute to the development of HCC [2]. Although diagnostic techniques have advanced, approximately 70% of cases of HCC are diagnosed at middle and late stages, missing the opportunity for radical surgery. In addition, the recurrence rate of liver cancer after surgery is approximately 70%, and surgery cannot be repeated in such cases [3]. However, there has been notable improvement in the survival of patients with advanced HCC since 2017 [4].

Recently, the combination of immune checkpoint inhibitors (ICIs) and kinase inhibitors emerged as a new therapeutic strategy for advanced HCC. ICIs, consisting of programmed death-1 (PD-1) and programmed death ligand-1 (PD-L1) antibodies, are new treatment modalities with clinical benefits in patients with HCC. PD-1 inhibitors include pembrolizumab, camrelizumab, tislelizumab, and nivolumab, whereas PD-L1 inhibitors include atezolizumab, durvalumab, and envafolimab. Kinase inhibitors include apatinib, sorafenib, lenvatinib, and regorafenib. In a real-world study of patients with unresectable HCC, the combination of lenvatinib and PD-1 inhibitors prolonged progression-free survival (PFS) and overall survival (OS). Median PFS and OS were 6.9 (95% confidence interval [CI] = 6.0–7.9) and 17.8 months (95% CI = 14.0–21.6), respectively, and the objective response rate (ORR) and disease control rate (DCR) were 19.6% and 73.5%, respectively [5]. A retrospective study of elderly patients with HCC described the efficacy and safety of sorafenib or lenvatinib plus PD-1 inhibitors, with median PFS and OS of 4.6 and 17.0 months, respectively [6]. Another multicenter retrospective study of patients with advanced HCC indicated that the combination of regorafenib and PD-1 inhibitors improved ORR and DCR and extended PFS and OS [7]. Similarly, in the CARES-310 study, camrelizumab plus rivoceranib prolonged PFS (5.6 months [95% CI = 5.5–6.3]) and OS (22.1 months [95% CI = 19.1–27.2]), whereas the ORR was only 25% in unresectable HCC [8]. ICI and kinase inhibitor combination treatment has displayed promise clinically, but their efficacy remains limited [9]. In summary, although the ICI/kinase inhibitor treatment significantly improved PFS and OS in advanced HCC, the survival benefit has been limited to a small portion of the population. Therefore, there is in urgent need to identify the patients who will benefit from new therapeutic approaches.

The study aimed to identify novel predictors of clinical outcomes for patients with advanced HCC who received ICI/kinase inhibitor therapy to improve survival outcomes.

## Methods and materials

### Patients

In this present study, patients with advanced HCC who received combination treatment with ICIs and kinase inhibitors at Ningbo Medical Center Lihuili Hospital between January 2020 and December 2023 were enrolled. Retrospective clinical data were collected independently by two oncologists. The inclusion criteria were as follows: age > 18 years, confirmation of HCC by pathology or clinical diagnosis (imaging and alpha-fetoprotein [AFP]); diagnosis of advanced HCC based on the China liver cancer staging criteria; Eastern Cooperative Oncology Group performance status (ECOG PS) ≤ 2; synchronous treatment with one anti-PD1/anti-PD-L1 inhibitor and one kinase inhibitor; peripheral blood testing was performed within 7 working days before treatment initiation; and the presence of at least one measured lesion according to the Response Evaluation Criteria in Solid Tumors (RECIST) 1.1 [10]. Meanwhile, the exclusion criteria were as follows: body mass index > 30; presence of other malignant tumors; presence of immune diseases prior to systemic treatment; prior receipt of other immune biotherapies; a lack of adequate laboratory data and clinical information; and reducing blood fat treatment before body treatments.

Patients’ clinical information comprised the following variables: sex; age; ECOG PS; the extrahepatic metastasis status at baseline; the portal vein thrombosis (PVT) status; HCC treatment history; the hepatitis B virus (HBV) status; HBV DNA levels; Child–Pugh classification; the ICI and kinase inhibitor received; alpha-fetoprotein (AFP), albumin (ALB, g/L), triglyceride (TG, mmol/L), total cholesterol (TC, mmol/L), high-density lipoprotein (HDL, mmol/L), and low-density lipoprotein (LDL, mmol/L) levels; and neutrophil (×10^9^/L), lymphocyte (×10^9^/L), and platelet counts (×10^9^/L) within 7 working days before treatment initiation. The prognostic nutritional index (PNI) was defined using the following formula: (10 × ALB + 0.005 × absolute lymphocyte count). The HBV DNA status was based on the guidelines for the prevention and treatment of chronic hepatitis B (version 2022). Serum HBsAg levels lower than 1000 IU/mL were indicative of non-reactivation and non-infection, whereas levels higher than 1000 IU/mL or equal to 1000 IU/mL denoted reactivation [11].

The Ethics Committee of Ningbo Medical Center Lihuili Hospital (KY2024SL072-01) approved this study, and all patients or their guardians provided informed consent before enrollment according to the Declaration of Helsinki (as revised in 2013).

### Evaluation of treatment responses

Based on RECIST 1.1, the efficacy of treatment was assessed by computed tomography or magnetic resonance imaging every 8–12 weeks until disease progression, and the treatment effect was categorized as progressive disease (PD), stable disease (SD), partial response (PR), and complete response (CR). The ORR was calculated as the sum of the CR and PR rates. Meanwhile, the DCR was calculated as the sum of the CR, PR, and SD rates.

According to RECIST 1.1, PFS was defined as the duration from the initiation of anti-PD1/anti-PD-L1 or kinase inhibitor treatment to tumor progression or death. OS was defined as the time from the initiation of anti-PD1/anti-PD-L1 or kinase inhibitor therapy until the date of the last follow-up or death. Anti-PD1/anti-PD-L1 therapy was started within 7 days of the initiation of kinase inhibitor treatment. Patient follow-up was conducted via telephone by May 2024.

### Evaluation of treatment toxicity

According to the National Cancer Institute Common Terminology Criteria for Adverse Events (version 5.0) [12], the adverse events (AEs) of the combination therapy were assessed every month.

### Statistical analysis

Clinical data were presented as proportions, medians, and ranges. Prognostic factors for PFS and OS were assessed by the Kaplan–Meier method. Hazard ratios (HRs) were estimated to identify independent factors via Cox regression survival. All tests were two-sided, and statistical significance was indicated by *P* < 0.05. Receiver operating characteristic (ROC) curves were drawn for the neutrophil-to-lymphocyte ratio (NLR), platelet-to-lymphocyte ratio (PLR), PNI, TG, TC, LDL, and HDL, and the proper cutoffs were determined using the Youden index. All statistical analyses were performed using SPSS 20.0 (IBM, Armonk, NY, USA) and R version 3.3.3 (R Foundation for Statistical Computing, Vienna, Austria).

## Results

### Patients’ characteristics

As presented in Table 1, 98 patients with advanced HCC who received combination treatment with anti-PD1/anti-PD-L1 immunotherapy and kinase inhibitors were identified. Of these patients, 87 were men, and 17 were older than 70 years. Most patients had ECOG PS 0–1 (80.6%) and Child–Pugh A (75.5%). Seventy-five patients had HBV infection, and 23 had a negative HBV status. Meanwhile, 62 patients had HBV DNA < 1000 IU/mL, and 36 had HBV DNA ≥ 1000 IU/mL. Sixty-one patients (62.2%) exhibited extrahepatic metastasis, and 41 (41.8%) had portal vein metastasis. Fifty-nine (60.2%) patients received combination therapy in the first-line setting. AFP levels exceeded 400 ng/mL in 44 patients (44.9%). In total, 95 patients (96.9%) received anti-PD-1 inhibitors, and 16 (16.3%) were treated with regorafenib.

**Table 1:** Clinical characteristics of patients (n = 98)

Characteristic	Patients, n (%)
Median age, years (range)	59.5 (28–90)
Age, years, n (%)	
< 70	81 (82.7)
≥ 70	17 (17.3)
Sex, n (%)	
Male	87 (88.8)
Female	11 (11.2)
ECOG PS	
0–1	79 (80.6)
2	19 (19.4)
HBV infection	
Positive	75(76.5)
Negative	23 (23.5)
HBV DNA status	
< 1000 IU/mL	62 (63.3)
≥ 1000 IU/mL	36 (36.7)
Child-Puge grade	
A	74 (75.5)
B	24 (24.5)
Extrahepatic metastasis	
Yes	61 (62.2)
No	37 (37.8)
Number of lines of prior systemic therapy	
0	59 (60.2)
1	39 (39.8)
Baseline serum AFP levels (ng/mL)	
< 400	54 (55.1)
≥ 400	44 (44.9)
PVT	
Yes	41 (41.8)
No	57 (58.2)
NLR	
< 3.225	55 (56.1)
≥ 3.225	43 (43.9)
PLR	
< 140.75	59 (60.2)
≥ 140.75	39 (39.8)
PNI	
< 37.25	25 (25.5)
≥ 37.25	73 (74.5)
HDL (mmol/L)	
< 1.105	32 (32.7)
≥ 1.105	66 (67.3)
LDL (mmol/L)	
< 2.145	34 (34.7)
≥ 2.145	64 (65.3)
TC (mmol/L)	
< 3.46	30 (30.6)
≥ 3.46	68 (69.4)
TG (mmol/L)	
< 0.705	26 (26.5)
≥ 0.705	72 (73.5)
ICI	
Anti-PD-1 antibody	95 (96.9)
Anti-PD-L1 antibody	3 (3.1)
Kinase inhibitor	
Sorafenib	19 (19.4)
Regorafenib	16 (16.3)
Lenvatinib	63 (64.3)

*ECOG PS* Eastern Cooperative Oncology Group performance status, *HBV* hepatitis B virus, *AFP* alpha-fetoprotein, *PVT* portal vein thrombosis, *NLR* neutrophil-to-lymphocyte ratio, *PLR* platelet-to-lymphocyte ratio, *PNI* prognostic nutritional index, *HDL* high-density lipoprotein, *LDL* low-density lipoprotein, *TC* total cholesterol, *TG* triglyceride, *ICI* immune checkpoint inhibitor

### Survival outcomes

**Fig. 1:**
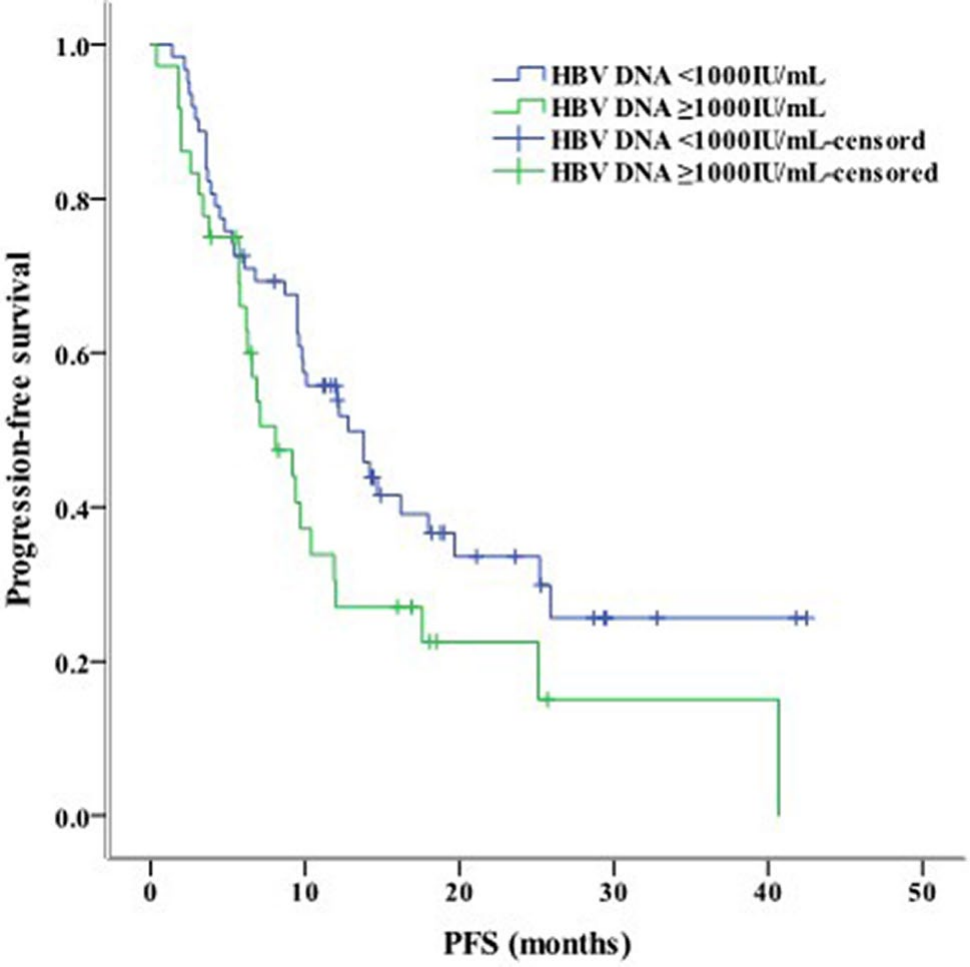
Kaplan–Meier curve analyses of PFS among patients with hepatocellular carcinoma accoding to the HBV DNA copy number. *PFS* progression-free survival, *HBV* hepatitis B virus

Median PFS was 10.1 months (95% confidence interval [CI] = 8.10–12.10), and median OS was 17.2 months (95% CI = 12.29–22.11). HBV infection and the number of lines of immunotherapy were not related to PFS and OS. However, the HBV DNA status was obviously relevant for PFS (*P* = 0.044, Fig. 1).

**Fig. 2:**
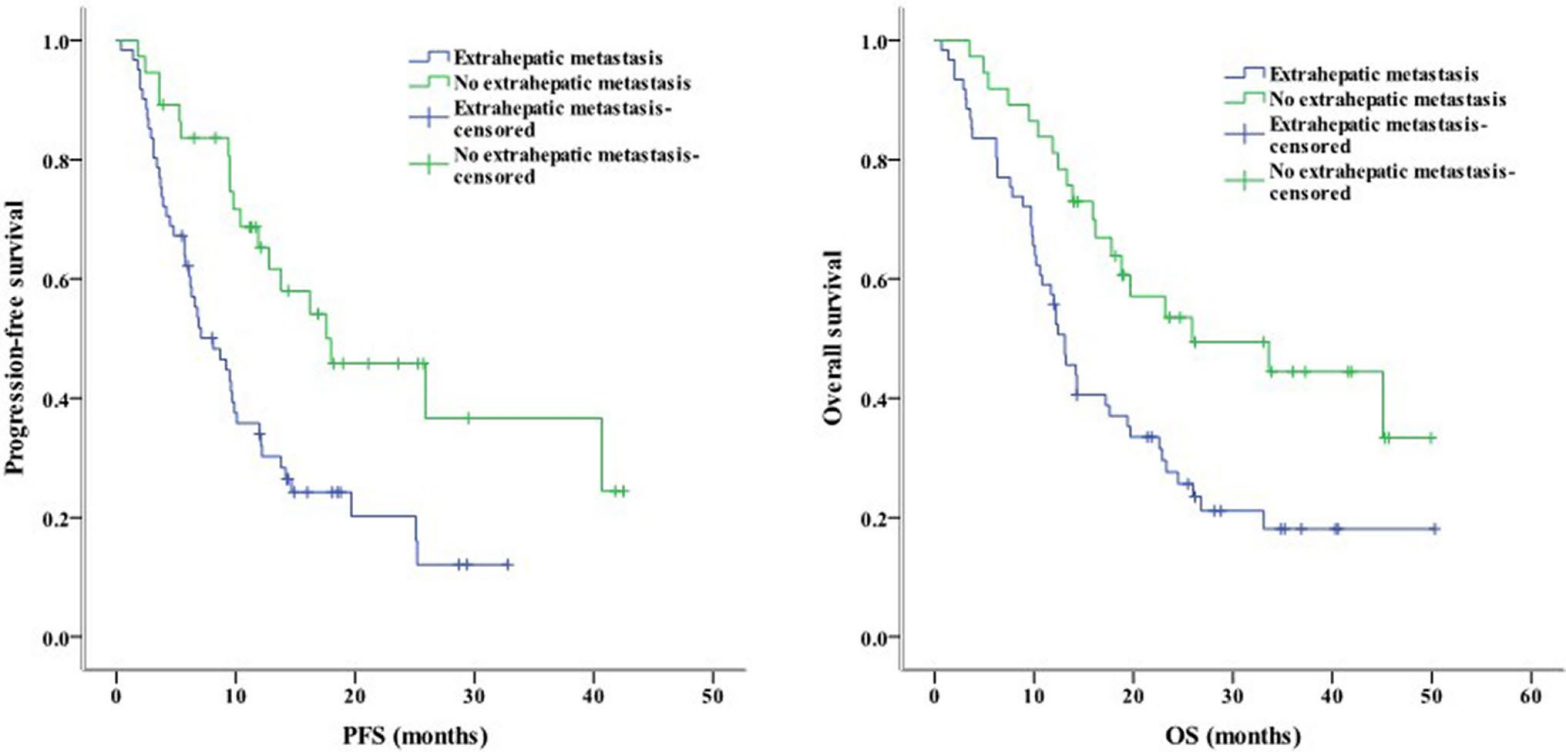
Kaplan–Meier curve analyses of PFS and OS among patients with hepatocelluar carcinoma according to the extrahepatic metastasis status. *PFS* progression-free survival, *OS* overall survival

**Fig. 3:**
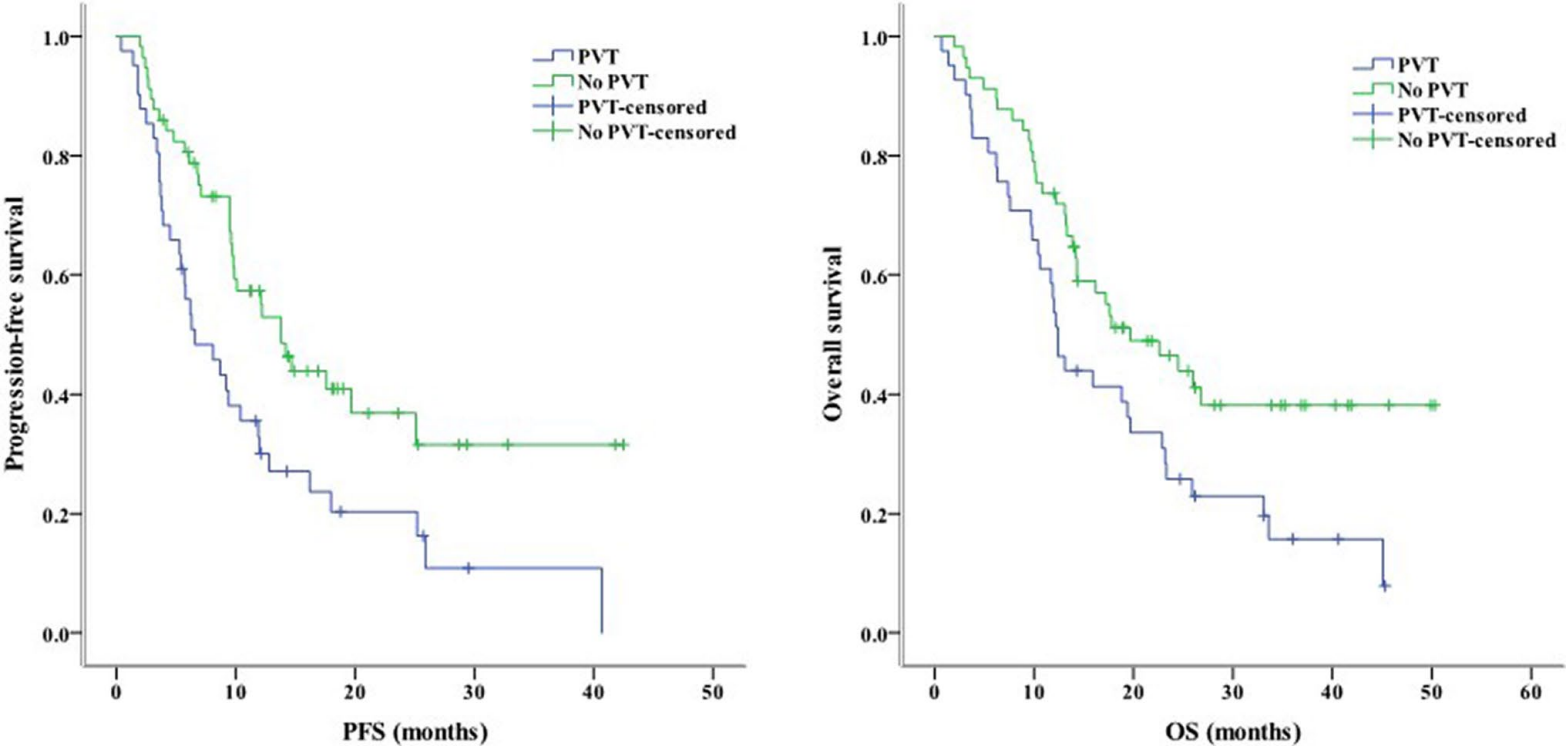
Kaplan–Meier curve analyses of PFS and OS among patients with hepatocelluar carcinoma according to the PVT status. PDamong patients with hepatocelluar carcinoma according to the PVT status. *PFS* progression-free survival, *OS* overall survival, *PVT* portal vien thrombosis

**Fig. 4:**
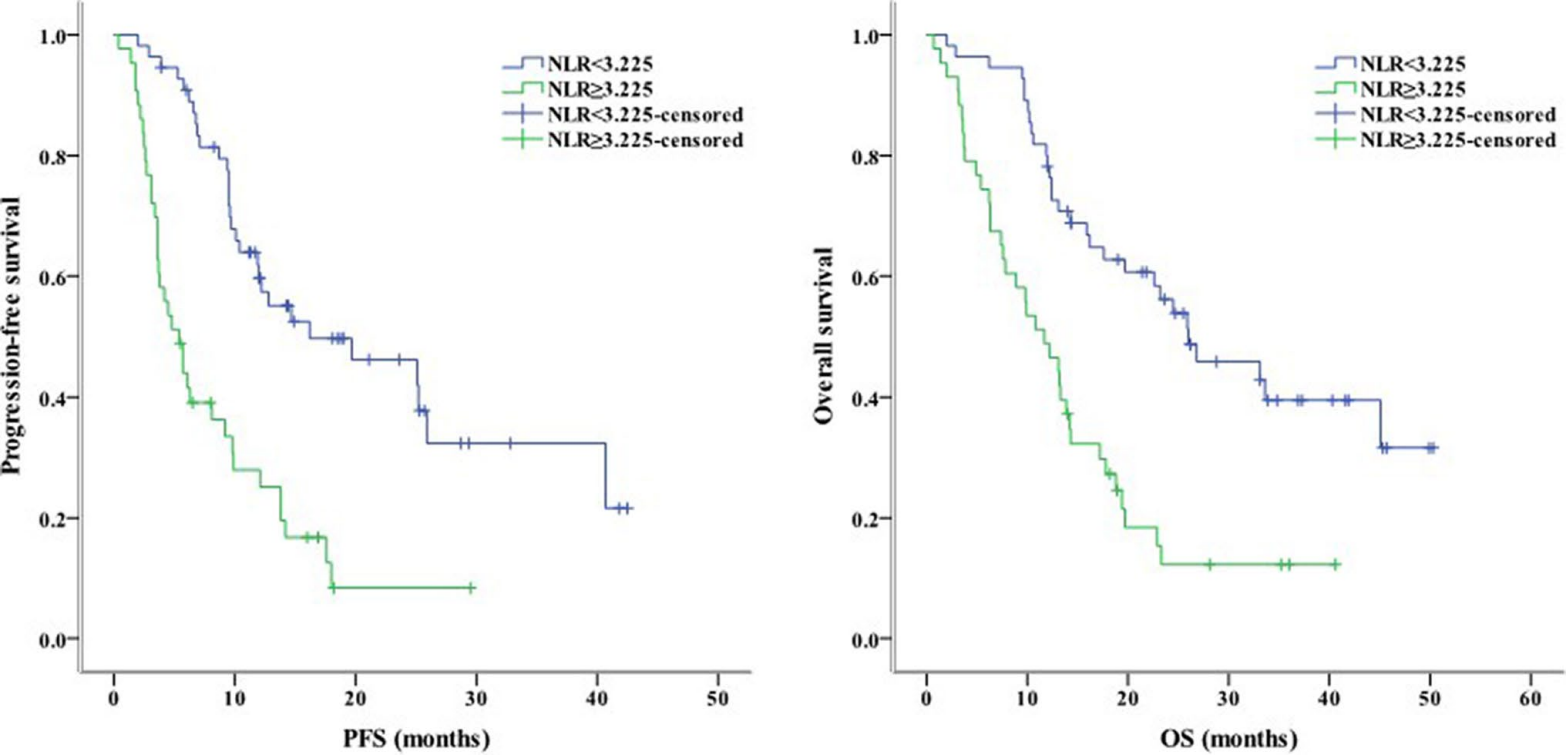
Kaplan–Meier curve analyses of PFS and OS among patients with hepatocelluar carcinoma according to NLR. *PFS* progression-free survival, *OS* overall survival, *NLR* neutrophil-to-lymphocyte ratio

**Fig. 5:**
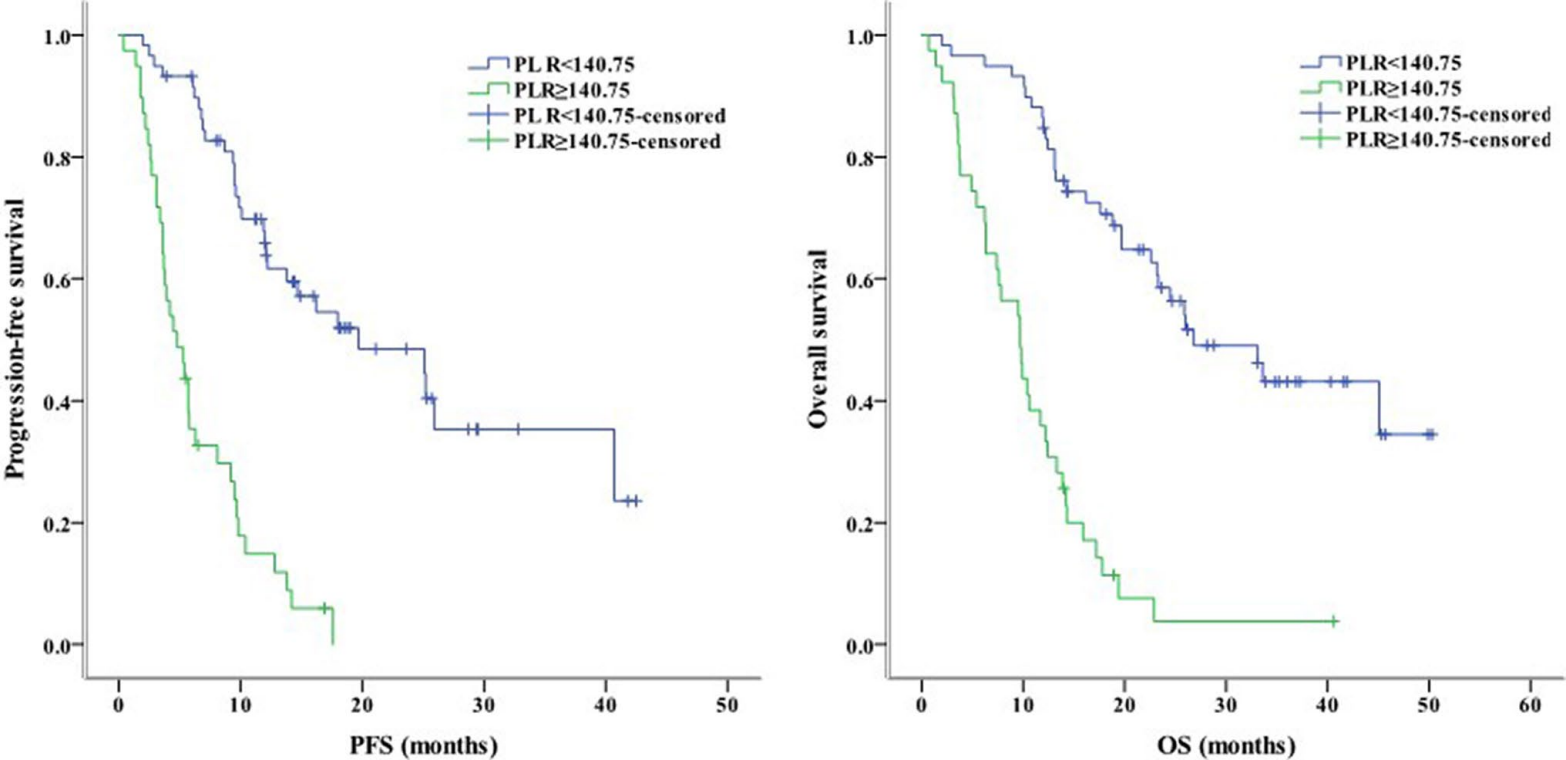
Kaplan–Meier curve analyses of PFS and OS among patients with hepatocelluar carcinoma according to PLR. *PFS* progression-free survival, *OS* overall survival, *PLR* platelet-to-lymphocyte ratio

**Fig. 6:**
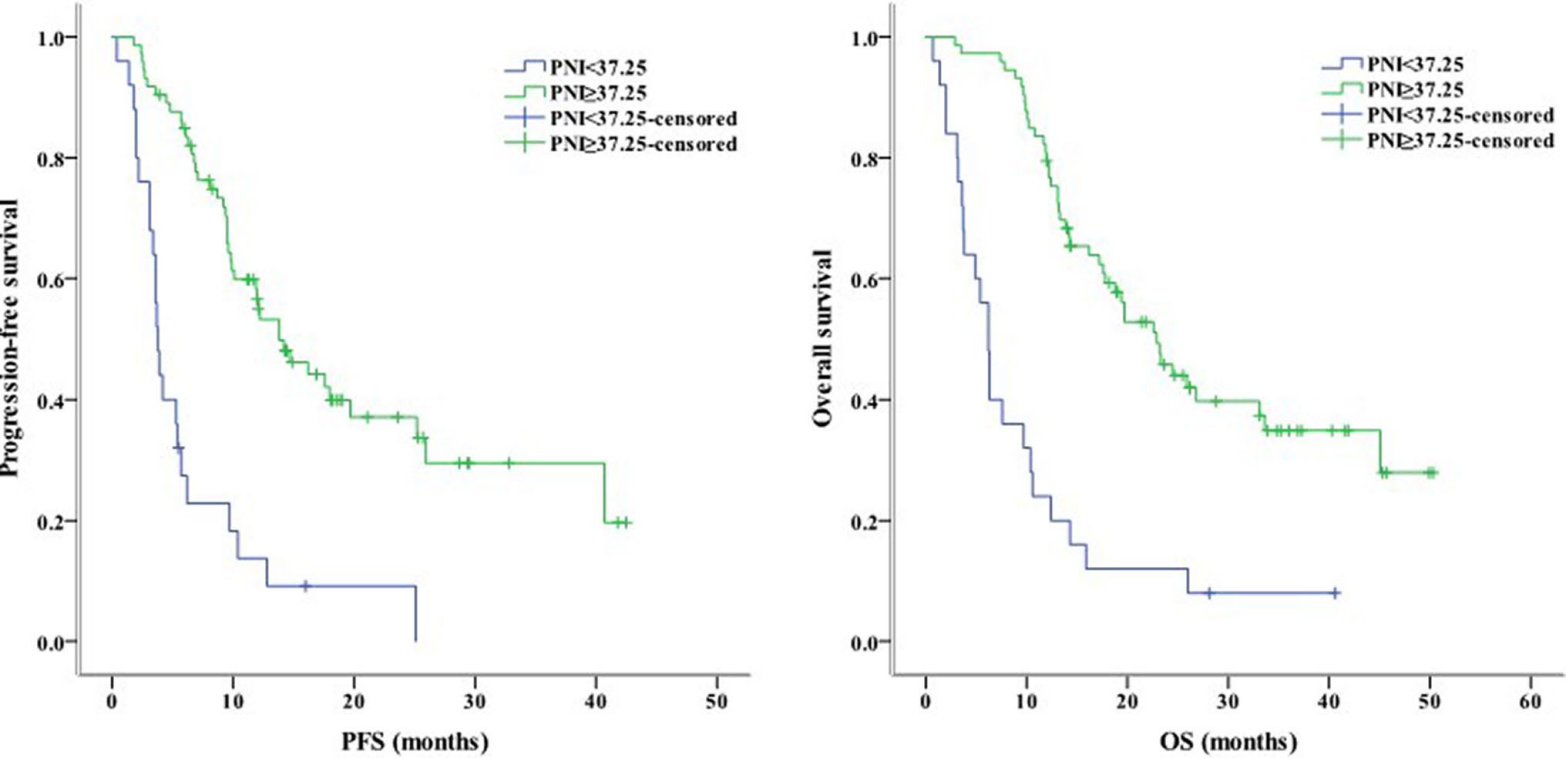
Kaplan–Meier curve analyses of PFS and OS among patients with hepatocelluar carcinoma according to PNI. *PFS* progression-free survival, *OS* overall survival, *PNI* prognostic nutritional index

**Fig. 7:**
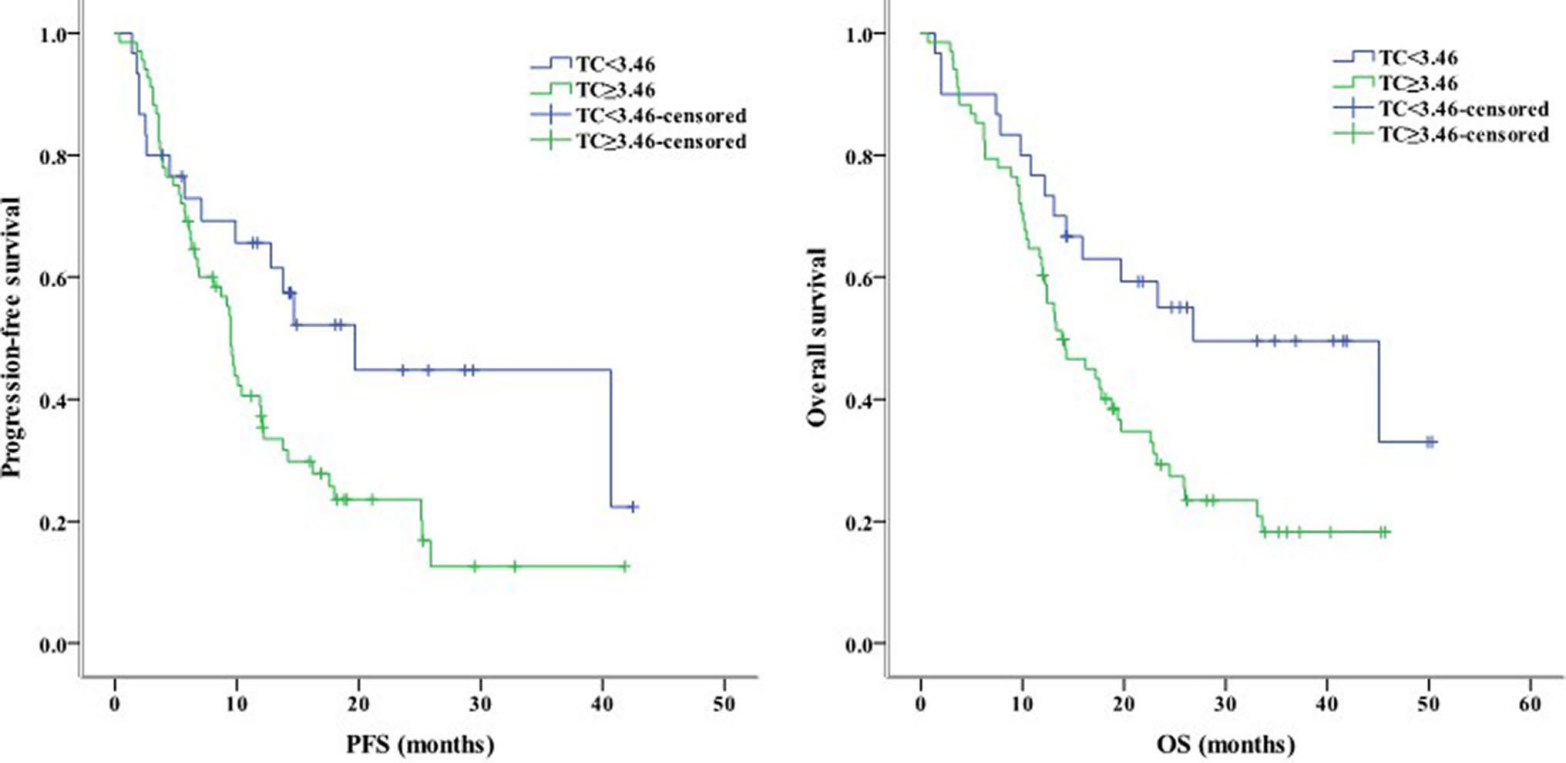
Kaplan–Meier curve analyses of PFS and OS among patients with hepatocelluar carcinoma according to the TC level. *PFS* progression-free survival, *OS* overall survival, *TC* total cholesterol

**Fig. 8:**
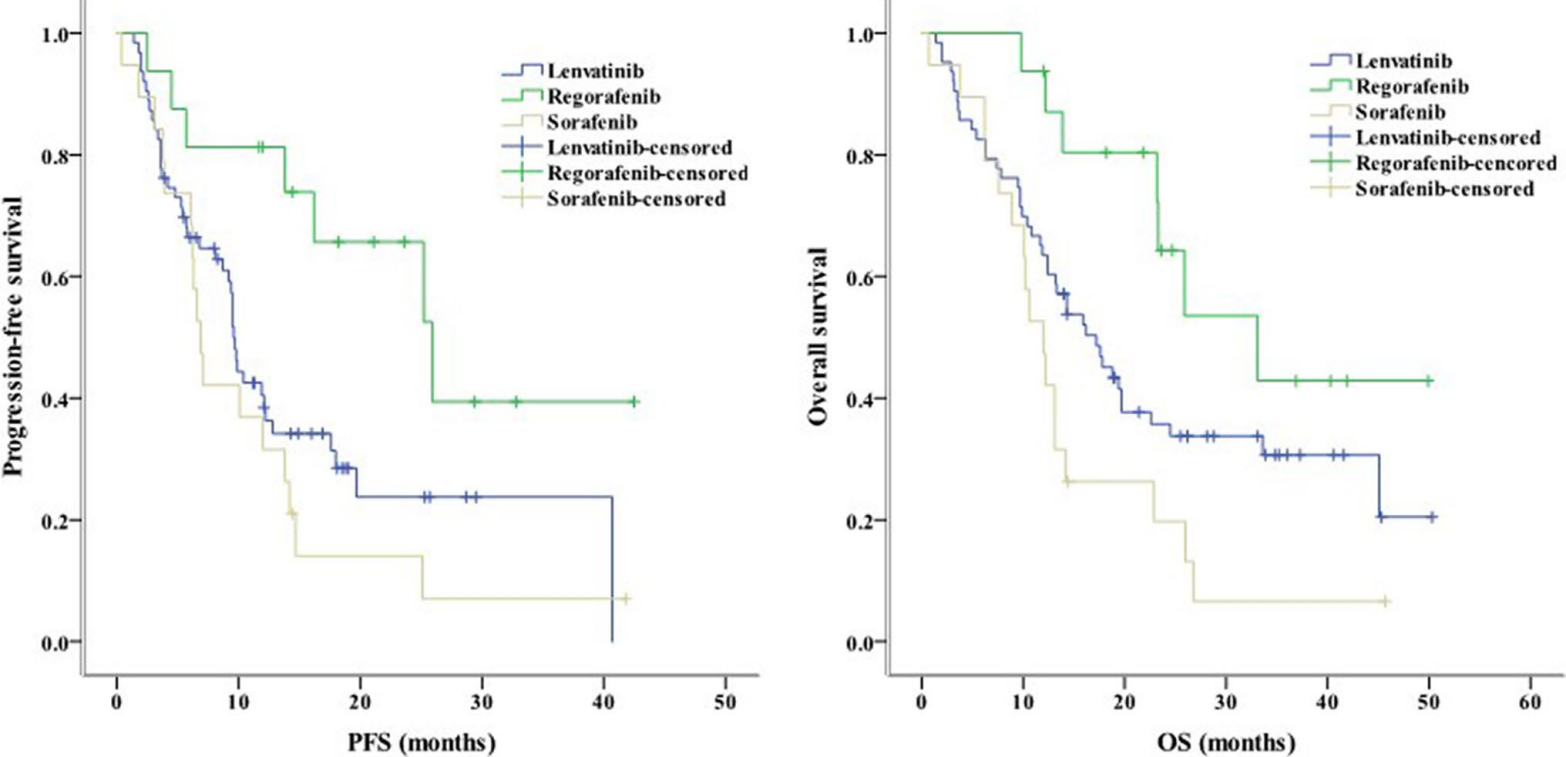
Kaplan–Meier curve analyses of PFS and OS among patients with hepatocelluar carcinoma according to the kinase inhibitors received. *PFS* progression-free survival, *OS* overall survival

Concerning clinical characteristics, univariate analysis identified ECOG PS 2 as a prognostic factor for worse PFS (*P* < 0.001) and OS (*P* = 0.003). Meanwhile, the absence of extrahepatic metastasis was linked to longer PFS (*P* = 0.001) and OS (*P* = 0.004, Fig. 2). The presence of PVT was associated with shorter PFS (*P* = 0.005) and OS (*P* = 0.016, Fig. 3). Moreover, PFS (*P* = 0.012) and OS (*P* = 0.007) were longer in patients with AFP < 400 ng/mL than in those with AFP ≥ 400 ng/mL. NLR ≥ 3.225, PLR ≥ 140.75, PNI < 37.25, and TC ≥ 3.46 mmol/L were associated with poor PFS and OS (Figs. 4, 5, 6, 7). Regarding the Child–Pugh classification, 98 patients were classified into class A or class B. Child–Pugh class A was associated with better PFS (*P* = 0.009) and OS (*P* = 0.001). Meanwhile, PFS significantly differed among the kinase inhibitors used (*P* = 0.014), and the receipt of regorafenib was associated with longer OS (*P* = 0.007, Fig. 8). However, the number of lines of prior systemic therapy, HDL, LDL, and TG were not significantly associated with PFS and OS (Tables 2, 3).

**Table 2:** Univariate and multivariate analyses for progression-free survival of all advanced HCC patients

Clinical variable	N	PFS univariate			PFS multivariate		
		Median (months)	95% CI	*P* value	HR	95% CI	*P* value
Age, < 70 years	81	10.1	7.52–12.68	0.937			
Sex, male	87	11.9	9.15–14.65	0.975			
ECOG PS				**0.000**	1.304	0.660–2.578	0.445
0–1	79	12.2	7.85–16.55				
2	19	3.9	1.48–6.32				
HBV infection				0.459			
Positive	75	10.4	7.79–13.01				
Negative	23	9.8	8.49–11.12				
HBV DNA status				**0.044**	0.784	0.452–1.363	0.389
< 1000 IU/mL	62	12.8	8.37–17.23				
≥ 1000 IU/mL	36	8.1	4.67–11.53				
Child–Pugh classification				**0.009**	0.994	0.508–1.944	0.987
A	74	12.8	8.62–16.98				
B	24	4.5	2.10–6.90				
Extrahepatic metastasis				**0.001**	0.311	0.164–0.589	**0.000**
Yes	61	8.1	4.97–11.24				
No	37	17.6	7.39–27.81				
Number of lines of prior systemic therapy				0.346			
0	59	9.5	7.82–11.18				
1	39	13.8	10.64–16.96				
Baseline serum AFP levels (ng/mL)				**0.012**	0.922	0.523–1.625	0.779
< 400	54	14.7	9.32–20.08				
≥ 400	44	6.9	3.54–10.26				
PVT				**0.005**	0.485	0.278–0.846	**0.011**
Yes	41	6.6	3.08–10.12				
No	57	13.8	10.61–16.99				
NLR				**0.000**	1.936	1.038–3.612	**0.038**
< 3.225	55	16.2	2.77–29.63				
≥ 3.225	43	5.4	3.51–7.29				
PLR				**0.000**	3.495	1.693–7.213	**0.001**
< 140.75	59	19.7	7.69–31.71				
≥ 140.75	39	4.8	2.97–6.64				
PNI				**0.000**	0.439	0.218–0.885	**0.021**
< 37.25	25	3.8	3.31–4.29				
≥ 37.25	73	13.8	9.72–17.88				
HDL (mmol/L)				0.355			
< 1.105	32	12.8	6.13–19.47				
≥ 1.105	66	9.8	7.54–12.06				
LDL (mmol/L)				0.155			
< 2.145	34	14.2	5.88–22.52				
≥ 2.145	64	9.7	9.18–10.22				
TC (mmol/L)				**0.030**	1.278	0.680–2.402	0.446
< 3.46	30	19.7	9.70–29.70				
≥ 3.46	68	9.5	8.86–10.14				
TG (mmol/L)				0.847			
< 0.705	26	9.4	0.00–20.54				
≥ 0.705	72	10.1	7.48–12.72				
ICI				0.393			
Anti-PD-1	95	10.1	7.57–12.63				
Anti-PD-L1	3	3.8	3.16–4.44				
Kinase inhibitor				**0.014**	1.032	0.738–1.442	0.855
Sorafenib	19	6.9	5.76–8.04				
Regorafenib	16	25.9	14.12–37.68				
Lenvatinib	63	9.6	9.10–10.10				

Bold values indicate the *p* value < 0.05

*ECOG PS* Eastern Cooperative Oncology Group performance status, *HBV* hepatitis B virus, *AFP* alpha-fetoprotein, *PVT* portal vein thrombosis, *NLR* neutrophil-to-lymphocyte ratio, *PLR* platelet-to-lymphocyte ratio, *PNI* prognostic nutritional index, *HDL* high-density lipoprotein, *LDL* low-density lipoprotein, *TC* total cholesterol, *TG* triglyceride, *ICI* immune checkpoint inhibitor

**Table 3:** Univariate and multivariate analyses for overall survival of all advanced HCC patients

Clinical variable	N	OS univariate			OS multivariate		
		Median (months)	95% CI	*P* value	HR	95% CI	*P* value
Age, < 70 years	81	17.8	12.47–23.13	0.722			
Sex, male	87	16.2	11.05–21.35	0.723			
ECOG PS				**0.003**	0.850	0.443–1.632	0.626
0–1	79	19.4	12.43–26.38				
2	19	10.6	3.78–17.43				
HBV infection				0.308			
Positive	75	19.4	11.40–27.40				
Negative	23	14.2	10.31–18.09				
HBV DNA status				0.136			
< 1000 IU/mL	62	19.7	12.46–26.94				
≥ 1000 IU/mL	36	12.2	10.58–13.82				
Child-Puge grade				**0.001**	1.682	0.892–3.171	0.108
A	74	19.7	14.53–24.87				
B	24	9.8	4.28–15.32				
Extrahepatic metastasis				**0.004**	0.603	0.333–1.091	0.094
Yes	61	13.1	10.76–15.44				
No	37	25.9	6.58–45.22				
Number of lines of prior systemic therapy				0.475			
0	59	14.3	6.87–21.73				
1	39	17.8	10.66–24.94				
Baseline serum AFP levels (ng/mL)				**0.007**	1.315	0.750–2.306	0.339
< 400	54	23.2	14.87–31.54				
≥ 400	44	12.2	9.58–14.82				
PVT				**0.016**	0.831	0.485–1.422	0.499
Yes	41	12.4	10.90–13.90				
No	57	19.7	10.94–28.46				
NLR				**0.000**	1.129	0.605–2.106	0.703
< 3.225	55	26.0	15.50–36.50				
≥ 3.225	43	11.7	7.46–15.94				
PLR				**0.000**	3.711	1.726–7.978	**0.001**
< 140.75	59	26.8	17.22–36.38				
≥ 140.75	39	9.7	7.13–12.27				
PNI				**0.000**	0.541	0.283–1.034	0.063
< 37.25	25	6.2	5.10–7.30				
≥ 37.25	73	22.9	17.86–27.94				
HDL (mmol/L)				0.412			
< 1.105	32	16.2	9.22–23.18				
≥ 1.105	66	17.6	11.20–24.00				
LDL (mmol/L)				0.095			
< 2.145	34	19.7	4.21–35.19				
≥ 2.145	64	14.3	9.50–19.10				
TC (mmol/L)				**0.016**	1.998	1.065–3.751	**0.031**
< 3.46	30	26.8	7.52–46.08				
≥ 3.46	68	13.9	9.72–18.08				
TG (mmol/L)				0.637			
< 0.705	26	19.7	5.05–34.35				
≥ 0.705	72	16.2	12.02–20.39				
ICI				0.548			
Anti-PD-1	95	17.2	12.89–21.51				
Anti-PD-L1	3	6.3	2.30–10.30				
Kinase inhibitor				**0.007**	1.121	0.810–1.551	0.490
Sorafenib	19	12.0	9.16–14.84				
Regorafenib	16	33.1	19.55–46.65				
Lenvatinib	63	17.2	12.21–22.19				

Bold values indicate the *p* value < 0.05

*ECOG PS* Eastern Cooperative Oncology Group performance status, *HBV* hepatitis B virus, *AFP* alpha-fetoprotein, *PVT* portal vein thrombosis, *NLR* neutrophil-to-lymphocyte ratio, *PLR* platelet-to-lymphocyte ratio, *PNI* prognostic nutritional index, *HDL* high-density lipoprotein, *LDL* low-density lipoprotein, *TC* total cholesterol, *TG* triglyceride, *ICI* immune checkpoint inhibitor

According to multivariate analysis, extrahepatic metastasis (*P* < 0.001; HR = 0.311; 95% CI = 0.164–0.589), PVT (*P* = 0.011; HR = 0.485; 95% CI = 0.278–0.846), NLR (*P* = 0.038; HR = 1.936; 95% CI = 1.038–3.612), PLR (*P* = 0.001; HR = 3.495; 95% CI = 1.693–7.213), and PNI (*P* = 0.021; HR = 0.439; 95% CI = 0.218–0.885) were predictive of PFS (Table 2). Conversely, PLR (*P* = 0.001; HR = 3.711; 95% CI = 1.726–7.978) and TC (*P* = 0.031; HR = 1.998, 95% CI = 1.065–3.751) were associated with OS (Table 3).

### Safety

All 98 patients were included in the assessment of treatment-related AEs. The combination of ICIs and kinase inhibitors was well tolerated, and no patients died. Rash was the most common AEs, and skin toxicity occurred in 34.5% of patients. Other AEs included hypothyroidism (23.5%), gastrointestinal toxicity (25.5%), nephrotoxicity (54.1%), hepatotoxicity (26.5%), and hypertension (73.5%). Twenty-one patients experienced grade 3–4 treatment-related AEs, including hypertension, nephrotoxicity, gastrointestinal toxicity and dermatologic toxicity (Table 4).

**Table 4:** treatment -related adverse events of ICI an kinase inhibitor

Adverse event	Any grade AEs,n (%)	Grade 3/4 AEs
Dermatologic toxicity	33 (34.5)	1 (1)
Hypothyroidism	23 (23.5)	0
Gastrointestinal toxicity	25 (25.5)	4 (4.1)
Blood toxicity	3 (3.1)	0
Nephrotoxicity	53 (54.1)	6 (6.1)
Hepatotoxicity	26 (26.5)	0
Hypertension	72 (73.5)	10 (10.2)
Angiocardiopathy	1 (1)	0
Respiratory and thoracic disorders	3 (3.1)	0

*ICI* immune checkpoint inhibitor, *AEs* adverse events

### Treatment response

The treatment response was CR, PR, SD, and PD in 0, 14 (14.3%), 70 (71.4%), and 14 patients (14.3%), respectively. The ORR and DCR were 14.3% and 85.7%, respectively.

## Discussion

In this retrospective study, we focused on the clinical characteristics of patients with advanced HCC to identify meaningful predictive factors for the outcomes of ICI/kinase inhibitor combination therapy. Although combination treatment is the most common strategy for advanced HCC, only a few patients benefit from such regimens. Furthermore, there is no consensus predictor for the efficacy of combination therapy. In our study, the ORR and DCR were 14.3% and 85.7%, respectively. The absence of extrahepatic metastasis, the absence of PVT, NLR < 3.225, PLR < 140.75, and PNI ≥ 37.25 were independent prognostic factors for PFS, whereas PLR < 140.75 and TC < 3.46 mmol/L were independent prognostic factors for OS.

The absence of extrahepatic metastasis and absence of PVT were identified as independent prognostic factors for PFS in patients with advanced HCC in this study. Consistently, prior research identified the absence of extrahepatic metastasis (*P* = 0.002; HR = 2.244; 95%CI = 1.365–3.689) and absence of PVT (*P* = 0.025; HR = 1.911; 95% CI = 1.377–2.572) as predictive of better prognoses [13, 14]. Wu et al*.* also found in a real-world study of patients who received lenvatinib and nivolumab for advanced HCC survival was worse in patients with PVT (*P* = 0.01; HR = 4.3; 95% CI = 1.5–12.8) [15]. These findings could be explained by the fact that PVT can lead to portal vein blockage, thereby affecting liver function [14]. Thus, the combination of ICIs and kinase inhibitors appears beneficial in patients with HCC patients but no extrahepatic metastasis or PVT.

NLR and PLR are easily assessed serum indices. In prior research, a lower NLR was linked to better survival among patients treated with the anti-PD-1 antibody sintilimab combined with regorafenib (*P* = 0.002; HR = 0.518; 95% CI = 0.257–0.955) [16]. In another study, a higher PLR was linked to worse tumor responses to ICI/kinase inhibitor combination therapy (*P* < 0.001; HR = 0.287; 95% CI = 0.143–0.575) [17]. The present study identified NLR as an independent factor for PFS, and PLR was predictive of both PFS and OS. In the tumor microenvironment, chronic inflammation is a well-known factor associated with malignant tumors, and it influences the outcomes of cancer [20]. In a cohort of 296 patients with unresectable HCC from 14 institutions who were treated with atezolizumab plus bevacizumab, NLR < 5.0 and PLR < 300 were correlated with better PFS [18]. Furthermore, there were no significant differences in the treatment response and AE rates between low and high NLR and PLR [18]. Compared with other predictors, NLR and PLR are objective, inexpensive, and clinically available parameters.

HCC is closely related to chronic inflammation, which is associated with ICI treatment [19, 20]. A few studies described the significance of ALB levels and lymphocyte counts in patients with malignancy [21–23]. Our study analyzed clinical data and found that pretreatment PNI was a low-cost, reliable, and independent index, as PNI < 37.25 predicted worse PFS (*P* = 0.021; HR = 0.439; 95% CI = 0.218–0.885). Furthermore, PNI has been reported to be an independent predictor in gastric cancer [22, 23], non-small cell lung cancer [21, 24], nasopharyngeal cancer [25], and HCC [20].

TC metabolism plays an important role in cancers, and it influences the tumor microenvironment by reprogramming immune cell function [26, 27]. Previous studies investigated the relationship between TC and prognosis. In patients with gastric cancer who received chemotherapy and PD-1 inhibitors, Tang et al*.* identified TC as a clinical notable biomarker for assessing survival [28]. In patients who received anlotinib for non-small cell lung cancer, multivariate analysis revealed that higher TC levels were associated with worse OS (*P* = 0.003; HR = 1.773; 95% CI = 1.213–2.592) [29]. Similarly, pretreatment TC > 3.46 mmol/L was associated with worse PFS and OS in the current study. Moreover, TC was a highlighted predictor for OS individually through ICI plus kinase inhibitor.

According to univariate analysis, patients’ survival was associated with HBV DNA levels, but not the HBV infection status, in this study. Specifically, high HBV DNA levels were associated with poor PFS (8.1 months; *P* = 0.044) and OS (12.2 months; *P* = 0.136) in patients with advanced HCC, in line with previous reports [30, 31]. Lei et al*.* stated that among patients with HCC who received kinase inhibitors alone or in combination with PD-1 inhibitors, PFS *(P* < 0.001) and OS (*P* = 0.001) were significantly better in the non-HBV reactivation group than in the HBV reactivation group [30]. Furthermore, among patients with HBV-related HCC who received PD-1 inhibitor therapy, 24 patients with HBV DNA < 1000 copies/mL had longer recurrence-free survival (*P* = 0.002; HR = 7.783) and OS (*P* < 0.001; HR = 6.699) than 20 patients with HBV DNA ≥ 1000 copies/mL [31]. This can be explained by the fact that HBV reactivation might damage the function, proliferation, and survival of natural killer cells [32]. Accordingly, HBV reactivation might be a risk factor for patients with HCC who received ICI and kinase inhibitor combination therapy.

Regorafenib appeared to be the best kinase inhibitor for advanced HCC in this study. Regorafenib can enhance the effect of immune checkpoint inhibitor by modulating the IFN-γ/NSDHL/SREBP1/TGF-β1 axis against HCC, and this treatment potently suppresses JAK1/2-STAT and MAPK signaling to attenuate IFNγ-induced PD-L1 expression.^[33.34]^ Moreover, regorafenib improved survival by increasing intratumoral CXCR3 + CD8 T cell infiltration and normalizing the cancer vasculature [35]. In this study, regorafenib was linked to prolonged median PFS (25.9 months; *P* = 0.014) and median OS (33.1 months; *P* = 0.007). However, multivariate analysis did not identify differences in PFS and OS among the kinase inhibitors used in this study. Thus, a randomized study of regorafenib plus ICI therapy is needed to identify the optimal combination therapy for advanced HCC.

### Limitations

This study had multiple limitations. First, this study was retrospective in nature, and the sample size was small. Thus, the existence of selection or statistical bias cannot be dismissed. Second, HCC was not proven by pathology in all cases. Third, no independent verification group checked the clinical application of the cutoffs. Fourth, there was no consensus regarding the sequence of ICI and kinase inhibitor therapy. Finally, different follow-up therapies might lead to different outcomes. Thus, future studies should seek to identify the optimal ICI/kinase inhibitor combination for patients with advanced HCC.

## Conclusion

This study identified extrahepatic metastasis, PVT, NLR, PLR, and PNI as independent clinical predictive factors for PFS and PLR and TC as independent predictors for OS in patients with advanced HCC who received combination therapy with ICIs and kinase inhibitors. Additionally, controlling HBV DNA levels before treatment is crucial for patients with advanced HCC and high HBV DNA levels. Although different combinations of ICIs and kinase inhibitors produced similar survival benefits, the findings suggested that regorafenib in combination with ICIs could improve survival outcomes in advanced HCC.

## Data Availability

The datasets generated during and/or analysed during the current study are available from the corresponding author upon reasonable request.
